# Investigating audible and ultrasonic noise in modern animal facilities

**DOI:** 10.12688/f1000research.111170.1

**Published:** 2022-06-14

**Authors:** Andrew Parker, Liane Hobson, Rasneer Bains, Sara Wells, Michael Bowl

**Affiliations:** 1 Mammalian Genetics Unit, MRC Harwell Institute, Harwell Oxford, UK; 2 Mary Lyon Centre at MRC Harwell, Harwell Science Campus, Oxford, UK; 3 UCL Ear Institute, University College London, London, UK

**Keywords:** Noise, Mouse, Welfare

## Abstract

**Background: **The environmental housing conditions of laboratory animals are important for both welfare and reliable, reproducible data. Guidelines currently exist for factors such as lighting cycles, temperature, humidity, and noise, however, for the latter the current guidelines may overlook important details. In the case of the most common laboratory species, the mouse, the range of frequencies they can hear is far higher than that of humans. The current guidelines briefly mention that ultrasonic (>20 kHz) frequencies can adversely affect mice, and that the acoustic environment should be checked, though no recommendations are provided relating to acceptable levels of ultrasonic noise.

**Methods: **To investigate the ultrasonic environment in a large mouse breeding facility (the Mary Lyon Centre at MRC Harwell), we compared two systems, the Hottinger Bruel and Kjaer PULSE sound analyser, and an Avisoft Bioacoustics system. Potential noise sources were selected; we used the PULSE system to undertake real-time Fourier analysis of noise up to 100 kHz, and the Avisoft system to record noise up to 125 kHz for later analysis. The microphones from both systems were positioned consistently at the same distance from the source and environmental conditions were identical. In order to investigate our result further, a third system, the AudioMoth (Open Acoustic Devices), was also used for recording. We used DeepSqueak software for most of the recording analysis and, in some cases, we also undertook further spectral analysis using RX8 (iZotope, USA).

**Results: **We found that both systems can detect a range of ultrasonic noise sources, and here discuss the benefits and limitations of each approach.

**Conclusions: **We conclude that measuring the acoustic environment of animal facilities, including ultrasonic frequencies that may adversely affect the animals housed, will contribute to minimising disruption to animal welfare and perturbations in scientific research.

## Introduction

Environmental factors, such as temperature, light cycle humidity and noise can have significant impacts on the physiology, behaviour and health of animals. ^1^^–^^5^ It is therefore key for both animal welfare and the quality and reproducibility of scientific findings that animals are housed in suitable conditions. Considerable research effort has focussed on determining the environmental conditions most appropriate for each species most commonly used in scientific studies; the basis of legislation regarding the use of animals in many countries is informed by these data. ^6^^–^^9^

The importance of the maintenance of certain environmental parameters within strict limits is not underestimated by those working with and caring for animals. Animal facilities have systems and procedures in place to prevent deviations from the limits outlined in animal use legislation, and have contingency plans in place in the event of a failure. ^9^^,^^10^ The conditions in which animals are housed are also stated in scientific publications and, if a system failure did occur during the study, the potential consequences are typically discussed therein. However, not all environmental factors are considered to be of equal importance to animal welfare and phenotype. In some cases, this is because current evidence does not show an impact of these environmental factors on welfare or phenotype. In others, there is emerging evidence of an impact, and potential reason for stricter control of these parameters, but research remains in relatively early stages and this has yet to transition fully to the legislature.

Mice are the most commonly used animals in scientific research. ^11^ Recent research has shown that the acoustic environment can have significant impacts on hearing, ^12^^–^^20^ anxiety, ^21^^–^^24^ cardiovascular function, ^25^^,^^26^ glucose tolerance ^27^^,^^28^ and reproductive success, ^29^^,^^30^ among others. The need for stricter control of the acoustic environment for mouse welfare and to ensure the production of high quality scientific data has been highlighted in previous reviews. ^31^^,^^32^ Other publications have made recommendations about how the acoustic environment might be controlled in animal housing facilities. ^31^^,^^33^ In response, governmental bodies and special interest groups released guidelines for monitoring and controlling the acoustic environment. ^6^^–^^8^^,^^10^ For example, the Home Office in the UK recommends that noise levels are assessed at a range of frequencies and that extraneous noise is minimised. ^10^

The current guidelines on monitoring the acoustic environment encourages animal housing facilities to monitor noise levels and highlights the importance of this for mouse welfare. However, as research continues to emerge on the impact of noise and the importance of ultrasonic vocalisations (USVs) in social communication in mice, ^34^^–^^38^ it is becoming increasingly clear that the current guidelines may lack the specificity required to minimise detrimental impacts on welfare and scientific quality. For example, there are no specifications regarding the length of surveys to assess noise levels or the frequencies of sound that should be considered in such surveys. This means that there is likely greater inter-facility variation in the acoustic environment compared to other factors known to impact welfare and phenotype. Facilities conducting longer surveys might be more likely to detect, and subsequently mitigate, periodic noises, and those assessing frequencies spanning the entire range audible to mice (1–100 kHz ^39^) could act to minimise extraneous noise from a greater range of sources. Any potential consequence of variation is difficult to detect though, because neither the methods used in sound surveys or noise levels are typically reported in publications.

Given the varied potential impacts of the acoustic environment on mouse welfare and phenotype, ^31^^,^^32^ it is crucial that methods are standardised between facilities. It is likely that facilities housing mice are primarily focussed on minimising extraneous noise audible to humans (human audible frequency range 0.02–20 kHz ^39^), as the majority of research investigating the impact of the acoustic environment on mice has examined the impacts of noise within this frequency range. ^31^^,^^32^ Further, those working with animals are able to detect such noise and note any possible impact on studies. Ultrasonic sounds (those with frequencies >20 kHz) have received less attention, because their impact on mice has not been investigated and they cannot be detected by humans. However, although mice have peak hearing sensitivity at 10–20 kHz, they also have increased hearing sensitivity at ~50 kHz, ^40^ so are able to hear ultrasonic sounds clearly. Indeed, the importance of ultrasonic vocalisations for social communication in mice is gaining research interest. ^34^^–^^38^ Taken together, this suggests that mouse welfare and phenotype could be impacted by extraneous ultrasonic noise in housing facilities.

The importance of implementing a framework for the maximum acceptable levels of audible and ultrasonic noise, as well as vibration within animal facilities has recently been proposed. ^41^ Whilst we do not report on vibration assessment here, we provide a comparison of ways that facilities may detect ultrasonic noise and show that mitigation is often easily and economically achieved.

Detection of ultrasonic noise is the first step in being able to assess the potential impact on mice and minimise extraneous noise. In this paper we evaluate the use of two different pieces of equipment (an Avisoft ultrasonic microphone and the Hottinger Brüel & Kjær (HBK) PULSE system) that can be used to monitor ultrasonic noise in animal housing facilities. We aim to raise awareness of the importance of standardising noise surveys between facilities and make recommendations for potential changes in legislation regarding control of the acoustic environment.

## Methods

### Selection of systems for testing ultrasonic noise

Multiple systems are capable of detecting and measuring ultrasonic sound. We selected two commercially available systems to identify sources of ultrasonic noise at the Mary Lyon Centre: 1) The Avisoft system - an Avisoft condenser microphone CM16/CMPA connected to an Avisoft UltraSoundGate 116Hb (Avisoft Bioacoustics, Berlin), and 2) the HBK PULSE system with a type 4939 ¼” measuring microphone attached to a high frequency 3110 processing module (Hottinger, Bruel & Kjaer UK).

These systems were selected as both can detect ultrasound and are of a very high quality. The Avisoft system is designed to monitor ultrasonic vocalisations (USVs) used in social communication and the PULSE system is a sound analysis platform designed for accurate noise and vibration measurement and analysis. Although the objective of both systems is to detect audible and ultrasonic noise, the intended purposes result in distinct differences between the two systems. The microphones differ in sensitivity: the Avisoft microphone CM16/CMPA (500 mV/Pa) is orders of magnitude higher than the HBK 4939 (4 mV/Pa), but by design, the HBK 4939 microphone has a much flatter frequency response, allowing single frequency calibration for accurate measurement. An AudioMoth (Open Acoustic Devices) full spectrum recorder was also used for one of the noise sources.

### Configuration used to record noise using the Avisoft Bioacoustics system

All recordings were completed using Avisoft Bioacoustics RECORDER software (Version 4.2.27) running on a Dell laptop computer. The sampling rate for recordings was 250 kHz, unless otherwise stated, enabling sounds with frequencies of up to 125 kHz to be detected. Recordings were stored as uncompressed 16-bit PCM .wav files.

### Configuration used to measure noise using the PULSE system

The PULSE system can be user configured from a number of optional hardware and software modules according to the requirements of the noise under investigation. Our system was configured to measure accurately in one second epochs up to 100 kHz; however, our system lacked the optional recording functionality, which can be added with a software licence, also we did not have the capability to measure vibration.

The type 4939 microphone has a frequency range of 4 Hz–100 kHz, and was calibrated before each measurement session using a 1 kHz test tone at 94 dB SPL from a pistonphone calibrator (Aco-Pacific type 511e). The flat response of this microphone allows single frequency calibration to be accurate across the stated frequency range. The A-weighting scale, normally used for noise assessments concerning humans, would not have been suitable as it only covers the acoustic range. Therefore, linear (unweighted) measurements were used. Fast Fourier Transform (FFT) analysis was performed in real time using the PULSE type 7700 sound analyser software running on a Hewlett Packard laptop computer. The FFT analyser was set to measure 6400 lines spanning the frequency range of 0 Hz–100 kHz, and stored measurements in a multi-buffer that refreshed every second. Whilst noise levels were analysed over this range, frequencies lower than 4 Hz were ignored due to the frequency range of the microphone. All sound pressure levels measured are in dB SPL (dB re. 20 μPa).

### Configuration used to record noise using the AudioMoth

The AudioMoth is a standalone full spectrum recorder; 16-bit PCM.wav files were written directly to a removable SanDisk microSD card. A sample rate of 384 kHz was used, enabling recording of noise up to 192 kHz, and the gain was set to high.

### Recording and measurement of potential sources of extraneous ultrasonic noise

Multiple potential sources of extraneous ultrasonic noise were identified in the Mary Lyon Centre. Possible sources that were considered included computers, air ventilation systems, ventilated hoods and changing station cabinets, equipment/procedures involving contact between two metal surfaces, walkie-talkie radios and light sources, among others. This is not a report of all the measurements taken, rather a comparison of the output of both systems to different types of potential ultrasonic noise sources: ceiling lights, commercially available walkie-talkie radios, and, contact between metal surfaces.

Unless stated otherwise, for data collection, microphones were positioned both inside a closed individually ventilated cage (IVC) (Tecniplast IVC 1284L) or on a tripod outside of an IVC. This enabled us to evaluate whether the mice would be exposed to the noise when housed in an IVC, such as exposure occurring under normal housing conditions, or only when removed from the IVC (e.g. during cage cleaning). For recordings taken inside the cage, the microphones associated with each system were inserted through holes made in the IVC, a PVC grommet was used to maintain an airtight seal. For recordings/measurements taken outside of the cage, the microphones were placed horizontally in a tripod stand. The microphone was positioned facing the noise source under investigation, at a distance of 20 cm.

### Analysing recordings of noise sources

The .wav files resulting from recordings made using the Avisoft system were high-pass filtered at 10 kHz and processed using DeepSqueak (version 2.6.0). All neural networks available on this version of DeepSqueak were applied to detect ultrasonic frequencies within the recordings and the default settings for detections were used. Measures of frequency and spectrograms of the structure of sounds were taken from DeepSqueak directly. Spectral analysis of the Avisoft recordings and the AudioMoth recordings was also performed in iZotope RX8.

### Data presentation

Data from the recordings from the Avisoft system, and measurements from the PULSE system are both presented in spectrogram form. These plot time against frequency, with the level/amplitude of the noise being denoted by a colour gradient scale adjacent to individual plots; however, only the PULSE system displays a calibrated measurement of noise in dB SPL. Spectrograms from the two systems are not directly comparable, but are presented to highlight the output from each system in response to different noise sources. The DeepSqueak spectrogram plots frequency vertically and time horizontally, whereas the PULSE spectrogram does the opposite. Another difference is the time scale: as mentioned the PULSE data is measured consistently in one-second epochs; the DeepSqueak system is a continuous recording and the timeframe used to present the data is in fractions of a second. However, the exact scale is variable due to software scaling dependent on the duration of noise. With a digital recording such as a.wav, there are numerous software options to analyse the data. In addition to the DeepSqueak software designed to automatically identify USVs within an audio recording, in one case iZotope RX8 was used. This analysis is again presented as a spectrogram, however the colours used to denote level are different from the other systems. The same spectral analysis could be performed using the open-source Audacity software.

## Results

Multiple sources of extraneous ultrasonic noise are likely to be present in any given animal facility. Rather than attempt to provide an exhaustive list of all potential noise sources, we have provided example sources for the three different types of ultrasonic noise identified in this study and evaluated the capacity of each system to detect and or measure each type of noise.

### Ceiling lights

**Figure 1.**
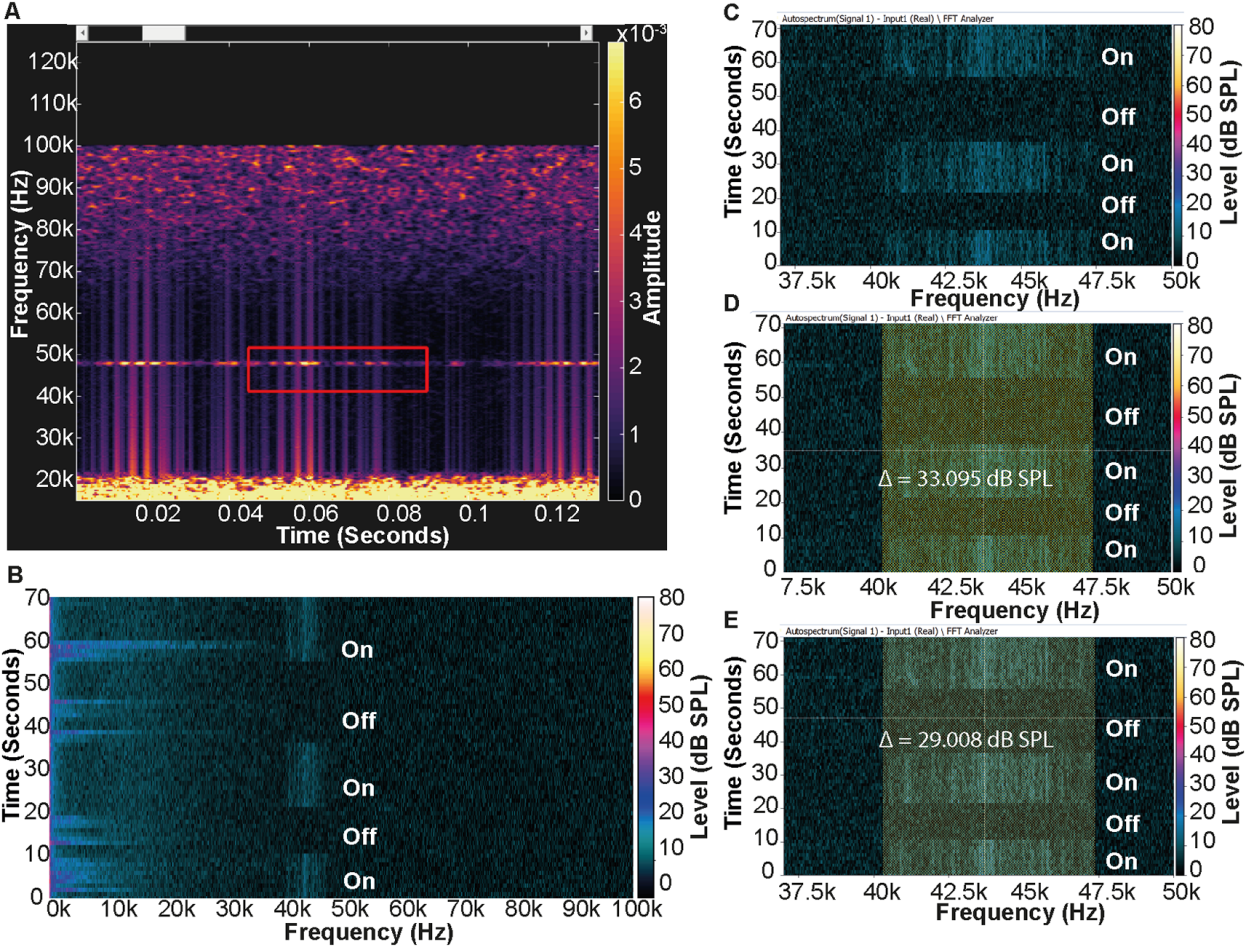
Spectrogram of the noise produced by the ceiling lights. A) Noise produced by the lights is highlighted in the red box. Spectrogram produced using DeepSqueak (2.6.0) ^36^. B) Full frequency range Fast Fourier Transform (FFT) autospectrum from the PULSE system highlighting the ultrasonic noise produced by the fluorescent ceiling lights. The periods when the lights were turned on or turned off are marked. The noise in the lower frequency bands was a result of background noise and movement in the adjacent corridor. C) FFT autospectrum from the PULSE system focused on the frequency range containing the ultrasound produced by the lights. D & E) The delta cursor, indicated in yellow, set to the frequencies spanned by the noise (40.3 kHz–47.4 kHz) measured 33.1 dB SPL when the lights were on (D) and 29 dB when off (E).

Some ceiling lights within the animal facility have been updated to include LEDs, but those that have not been updated produced continuous ultrasonic noise. To confirm that the lights were the source of the noise and to assess background noise level, the lights were turned on and off during tests ( Figure 1).

The Avisoft system detected low-level noise with a frequency just below 50 kHz ( Figure 1A). Consistent with this finding, measurements taken from recordings made by the PULSE system using a delta cursor showed that the lights produced ultrasonic noise with frequencies between 40.3 kHz and 47.4 kHz ( Figure 1B & C). Further measurements were taken using the PULSE system to confirm the source, and to calculate the sound pressure level using a delta cursor covering this frequency range. A level of 33.1 dB SPL was present in the delta cursor when the lights were on ( Figure 1D), compared to ~29 dB SPL when the lights were switched off ( Figure 1E). A change of 3 dB SPL equates to a doubling of sound pressure; this does not mean it is twice as loud but if it were audible noise, humans would be able to detect the change. Using the standard background noise correction calculation, we calculated that the lights were producing ultrasonic noise in the specified frequency range at a level of 30.8 dB SPL.

When measuring from inside the IVC, neither system detected any change from background levels, suggesting that the cage is sufficient to attenuate the noise and therefore mice will not be exposed to this sound under normal housing conditions. However, both systems detected the sound when positioned on a tripod outside of the IVC, so when removed from the cage during handling, or if housed in conventional caging in facilities without IVCs, this would be audible for mice.

### Walkie-Talkie radios

When using the Avisoft system, a noise related to the use of commercially available walkie-talkie radios was identified. The results were similar for multiple brands of radio, suggesting that this noise is inherent to this technology.

**Figure 2.**
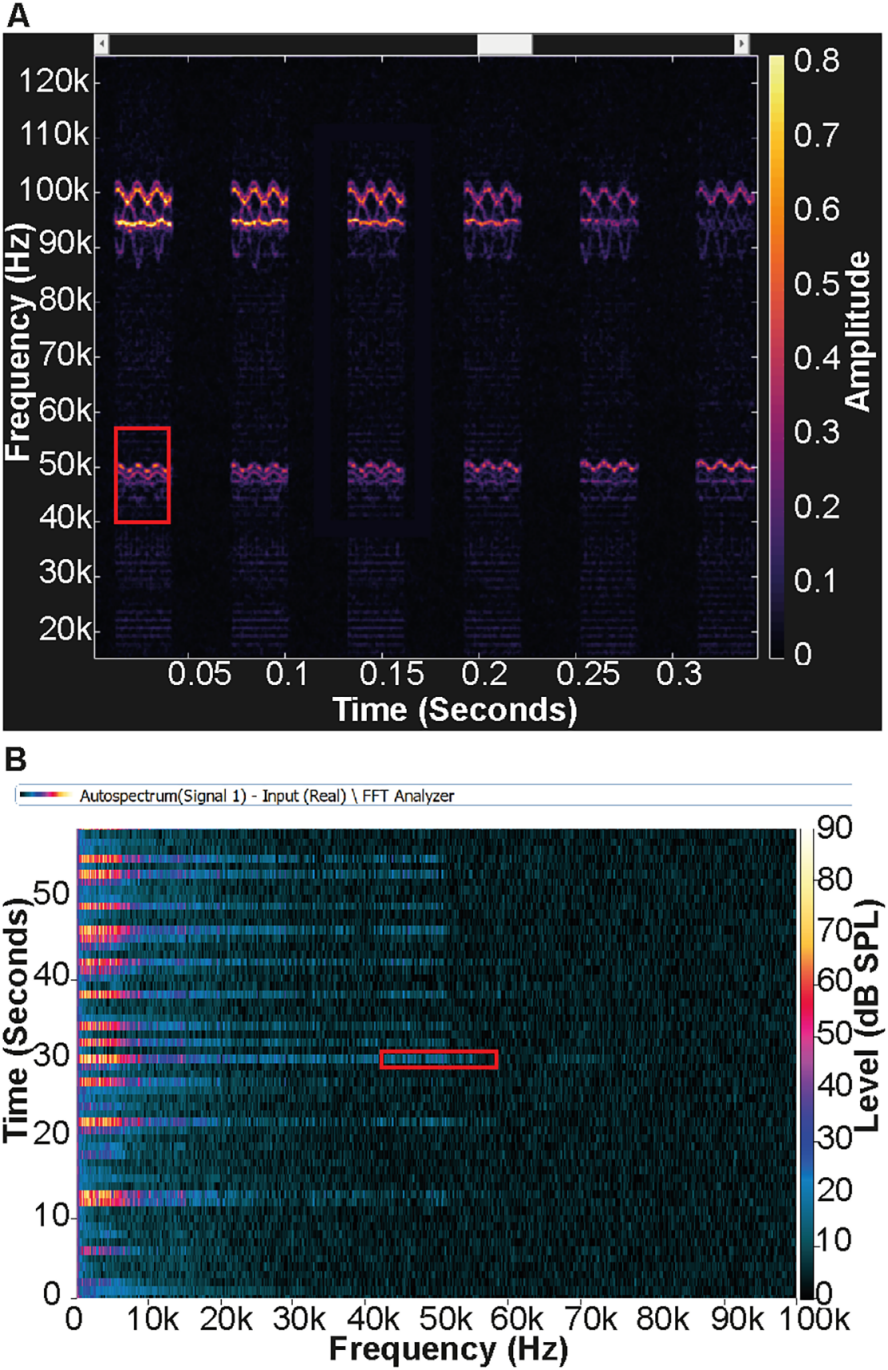
Spectrograms comparing the Avisoft and PULSE systems in relation to operation of walkie-talkie radios. A) The Avisoft system detected a multi component frequency modulated noise centred at approximately 50 kHz and 100 kHz. B) The PULSE system did not detect any noise at 100 kHz. The red boxes in (A) and (B) highlight comparable frequency ranges in each spectrogram. Whilst the PULSE system did measure a low-level noise in this range, it is likely related to harmonics of the higher-level audible beep in the 1–7 kHz range.

The Avisoft Bioacoustics system reliably detected the noise produced by the radios. The spectrograms produced by DeepSqueak indicated the noise was repetitive, highly transient and frequency modulated (FM) with multiple components. One element modulated at 48–50 kHz and another at 98–102 kHz ( Figure 2A, C). Both components would therefore potentially be audible to mice, ^39^ with the former component, like the ceiling lights, within the frequency range at which mice display an elevated sensitivity to ultrasonic sound. ^40^

In contrast, the PULSE system did not detect the noise from the walkie-talkie radios as efficiently. The PULSE system could detect a component centred on 50 kHz. The FFT autospectrum indicated low-level noise with frequency content spreading from 20 kHz to 50 kHz, coinciding with the audible beep produced by the operation of the radio ( Figure 2B). The frequency spread seen in the spectrogram suggests that this noise could be harmonic frequencies related to the high-level noise of the audible beep from the radio below 7 kHz. Interestingly, the PULSE system did not detect any noise in the 100 kHz range ( Figure 2B).

Further experiments were undertaken to investigate the disparity between the results obtained from the two systems. The Avisoft system detected this noise even when the microphone was housed within the IVC, at a distance of 10 metres and through a wall within the animal facility. In contrast, the noise could not be detected by the PULSE system outside of the IVC with the radio a short distance (20 cm) from the microphone. Ultrasonic sound is readily absorbed by surfaces and is very unlikely to pass through walls, therefore, this suggested that the DeepSqueak spectrogram was potentially displaying an artefact.

**Figure 3.**
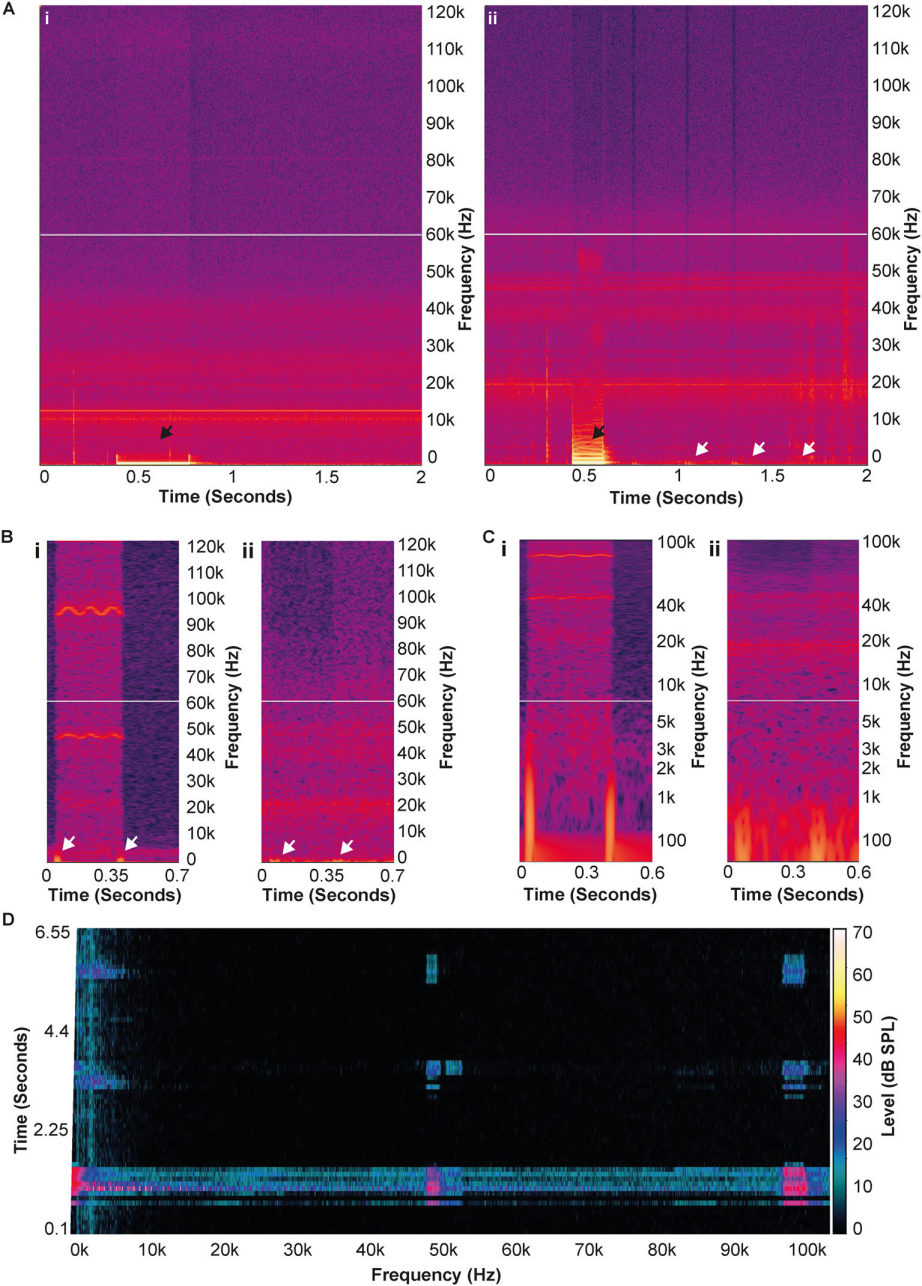
The walkie-talkie noise was likely radio frequencies (RF) interference. A) Spectrograms of the AudioMoth recordings of the radio being used in an isolated room (i), and in close proximity to an active monitor speaker (ii). The black arrows denote the audible beep produced when the transmit button was pressed. The white arrows in (ii) show the RF interference picked up by the active monitor speaker, while the absence of these in (i) shows that the noise did not arise from the walkie-talkie alone. B) Comparison of the spectrograms from the Avisoft recording (i) and the AudioMoth recording of the radio by the speaker (ii). The white arrows again denote the audible clicks in both recordings, which are not produced by the walkie-talkie itself (as shown in [Ai]); note the ultrasonic noise picked up the Avisoft system are bordered by these clicks. C) The same comparison as (B) using a logarithmic frequency scale to highlight the temporal similarities between the noise from the Avisoft system and the radio being operated in close proximity to the speaker. D) FFT spectrogram using the PULSE software to analyse the recording made by the Avisoft system. Noise can clearly be seen at the same frequencies that were originally detected by the Avisoft system, indicating that if the noise was not an artefact, the PULSE system would have also detected it.

Upon audio playback of the unfiltered .wav file recorded by the Avisoft system, it was apparent that some sort of interference was present in the recording. The audible beep from the radio could be heard, but a clicking sound was also present that was not heard when using the walkie-talkie at the time of recording. Spectral analysis of the unfiltered, full spectrum recording (iZotope RX8) showed that the clicking noise was concurrent with the higher frequency noise detected by DeepSqueak ( Figure 3Bi).

Walkie-talkie radios use FM radio frequencies (RF) in the MHz range. Ultrasonic sound propagates as a mechanical wave and requires a medium such as air to travel. Radio waves are electromagnetic waves, which can travel great distances and can easily pass through walls, as they do not require a medium to travel. As electromagnetic waves do not cause fluctuations in air pressure, they cannot be recorded using microphones in the normal way, however, poorly shielded microphones and cables can act as an aerial and pick up interference from RF sources. ^42^ It is possible that the extremely sensitive Avisoft CM16 microphone, or indeed the cable used could also potentially be acting as an aerial, and the spectrogram was showing this as an intermittent FM signal.

A third device, an AudioMoth (Open Acoustic Devices), was utilised in an attempt to determine if the noise detected by the Avisoft system was indeed due to RF interference. The AudioMoth is a full spectrum audio-logger which contains a Micro-Electromechanical Systems (MEMS) microphone, and is capable of producing full-spectrum recordings at a maximum sample rate of 384 kHz. By design, MEMS microphones are much less sensitive to RF or electromagnetic interference. Whilst the audible beep was picked up by the AudioMoth, the additional ultrasonic component was not ( Figure 3A, Bii and Cii). Operation of the radio in close proximity to a pair of active monitor speakers (Alesis, USA), produced a noise similar to ‘GSM Buzz’, the noise produced by RF interference with speaker coil/cables, that can sometimes be heard when a mobile (cell) phone is in use in close proximity. The spectrogram of this noise shows it occurs in a similar temporal pattern to that of the clicking noise seen in the Avisoft recording ( Figure 3C).

Furthermore, when the recording from the Avisoft microphone was used as the input for the PULSE analysis software, noise in both regions of 50 kHz and 100 kHz could be identified ( Figure 3D). Taken together, these findings strongly suggest that the noise picked up by the Avisoft system was in fact RF interference; therefore, it would not be audible to, or affect mice.

### Impact noise

**Figure 4.**
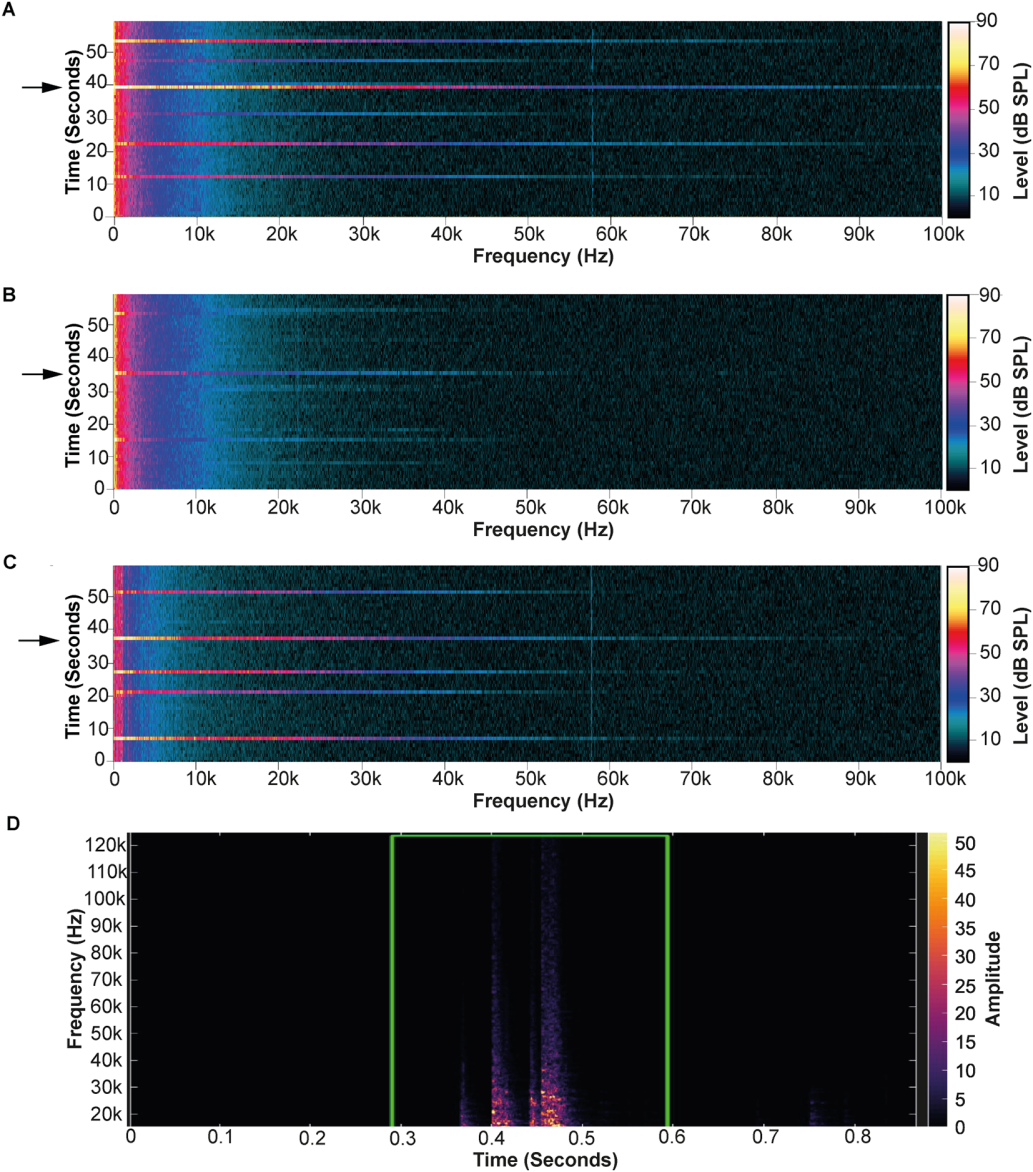
Measurements of the noise produced from forceps impacting surfaces. A) Fast Fourier Transform (FFT) autospectrum from the PULSE software showing the noise measured by dropping metal forceps on a metal LAF cabinet surface. B) Similar measurements taken using a neoprene mat as a noise control measure. C) Measurements from inside the modified individual ventilated cage (IVC) when the forceps were dropped on the metal surface of the LAF cabinet. The black arrows indicate the records used for analysis shown in Table 1, background readings in (A), (B) and (C) were taken from records where the forceps were not dropped. D) Spectrogram from DeepSqueak of the recording made outside the LAF cabinet with the Avisoft system to highlight that it was able to detect the noise. Again, a 10 kHz high-pass filter was used on the recording so much of the high-level sound in the human audible range is not displayed. However, the ultrasonic frequency content was similar to that measured by the PULSE system.

Perhaps the most common cause of noise within an animal facility are impacts, in particular those involving contact between metal objects. We detected high-level noise with a widespread frequency content when a pair of metal forceps, commonly used for checking copulatory plugs, were dropped from normal working height onto the metal surface of a laminar air flow (LAF) cabinet. Although relatively transient, this noise was detected using both systems when the microphones were placed on a tripod outside of an IVC. The spectrogram for the Avisoft system can be seen in Figure 4D.

In an attempt to approximate the exposure of a mouse to this noise during copulatory plug checks, the microphone for the PULSE system was positioned inside the LAF cabinet approximate to that of a mouse during this routine husbandry procedure. Given that it is not possible to perform repeated impacts in a uniform manner, and the fact that the highest levels are likely to have the greatest effect on the mice, measurements of sound pressure level from the highest level of impact were used. Background noise correction is not necessary for the calculation of the impact noise level; the measured sound pressure level was sufficiently high in each case to be equivalent to that of the impact. However, the level increase over background levels for each frequency band were used to highlight where mice may have heightened sensitivity to the sound due to normally low background levels. Sound pressure levels were also measured within the modified IVC inside the LAF cabinet to analyse noise audible to mice in the cage. The Avisoft system was not used inside the LAF cabinet as it could detect the sound from outside and is not able to measure sound pressure levels.

Measured noise levels for the different frequency ranges are shown in Table 1. At their peak, noise levels from the impact of the forceps on the base of the LAF cabinet reached a total of 112.11 dB SPL across the full frequency range of 4Hz–100 kHz ( Figure 4A and Table 1). The majority (112.03 dB SPL) was concentrated within the human audible range (20 Hz–20 kHz), ^39^ which includes the range of mouse peak hearing sensitivity (10 kHz–20 kHz), ^40^ although noise levels were highest below 10 kHz. In addition, very high-level noise was also detected at frequencies within the ultrasonic range (20 kHz–100 kHz) ( Figure 4A and Table 1). The impact noise caused an increase of 49.34 dB SPL relative to background noise, both across the range of mouse peak hearing sensitivity and the ultrasonic frequency range. This increase in sound pressure level relative to background across those frequencies was greater than that evident at frequencies <10 kHz (~26.1 dB SPL increase) ( Figure 4A and Table 1). This indicates that metal on metal impacts of this kind may expose mice to a substantial increase in noise across a wide range of frequencies audible to them, for which background levels of noise are typically very low (~45 dB SPL).

**Table 1.** Measurements of forceps impacts. Measurements of background noise levels, noise produced when dropping metal forceps in different circumstances as described, the difference between background and measured impact noise and the reduction when using a neoprene mat as a noise control measure. IVC: individual ventilated cage; BG: Background.

**Noise band**	**Frequency range**	**Metal (BG)**	**Metal**	**Difference**
Total	4 Hz–100 kHz	85.96 dB SPL	112.11 dB SPL	**26.15 dB SPL**
Human audible	20 Hz–20 kHz	85.91 dB SPL	112.03 dB SPL	**26.12 dB SPL**
Mouse peak	10 kHz–20 kHz	43.83 dB SPL	97.71 dB SPL	**53.88 dB SPL**
Ultrasonic	20 kHz–100 kHz	40.63 dB SPL	89.97 dB SPL	**49.34 dB SPL**
**Noise band**	**Frequency range**	**Neoprene (BG)**	**Neoprene**	**Difference**
Total	4 Hz–100 kHz	88.67 dB SPL	100.34 dB SPL	**11.67 dB SPL**
Human audible	20 Hz–20 kHz	87.87 dB SPL	100.36 dB SPL	**12.49 dB SPL**
Mouse peak	10 kHz–20 kHz	45.24 dB SPL	62.26 dB SPL	**17.02 dB SPL**
Ultrasonic	20 kHz–100 kHz	41.23 dB SPL	54.5 dB SPL	**13.27 dB SPL**
**Noise band**	**Frequency range**	**Reduction**		
Total	4 Hz–100 kHz	**14.48 dB SPL**		
Human audible	20 Hz–20 kHz	**13.63 dB SPL**		
Mouse peak	10 kHz–20 kHz	**36.86 dB SPL**		
Ultrasonic	20 kHz–100 kHz	**36.07 dB SPL**		
**Noise band**	**Frequency range**	**IVC (BG)**	**IVC**	**Difference**
Total	4 Hz–100 kHz	65.4 dB SPL	102.3 dB SPL	**36.9 dB SPL**
Human audible	20 Hz–20 kHz	79.7 dB SPL	104.1 dB SPL	**24.4 dB SPL**
Mouse peak	10 kHz–20 kHz	36 dB SPL	83.4 dB SPL	**47.4 dB SPL**
Ultrasonic	20 kHz–100 kHz	40.2 dB SPL	76.4 dB SPL	**36.2 dB SPL**

As these metal-on-metal impacts occur frequently in the animal unit, we repeated the test using conditions identical in every way, except that a neoprene mat was placed on the metal LAF cabinet base, to cushion the impact of the forceps. The neoprene mat prevented the metal-on-metal impact, resulting in a substantial attenuation of noise ( Figure 4B and Table 1), most importantly a relative reduction of ~36 dB SPL in both the ultrasonic and the mouse peak hearing sensitivity frequency ranges ( Figure 4B and Table 1).

Measurements taken from inside the IVC showed that the cage provided ~10 dB SPL attenuation across the 4 Hz–100 kHz range investigated; however, the metal on metal impact was of a sufficiently high level to be easily audible to mice inside the cage. The measurement in the ultrasonic range was still very high, an increase over background of 36.2 dB SPL ( Figure 4C and Table 1). These data combined with the findings from the ceiling lights, show that whilst the plastic cage is sufficient to shield mice from low-level ultrasonic noise, higher levels can still pass through.

## Discussion

In recent years, it has become increasingly apparent that exposure to noise, particularly high-level noise within the range audible to humans (20 Hz–20 kHz), can impact mouse welfare and influence multiple aspects of phenotype (see *e.g.* Refs. 15, 21, 22), thus potentially confounding studies. While current guidelines have been beneficial in encouraging the monitoring of the acoustic environment in animal housing facilities, they lack specificity, enabling variation between animal housing facilities. In this paper, we wanted to draw attention to the importance of monitoring ultrasonic noise sources, for two reasons in particular:

1. Ultrasonic noise is inaudible to humans, so those working with mice are unlikely to perceive the noise and take subsequent steps to mitigate it. By contrast, they are likely to take steps to minimise noise at frequencies audible to both humans and mice, preventing prolonged exposure to high level noise at these frequencies, which is known to be harmful to both humans and mice. ^15^^,^^21^^,^^22^
2. There is considerable evidence that animals can be detrimentally affected by noise within their audible range. ^25^^,^^43^ However, whilst we know that ultrasonic frequencies are audible to mice, ^39^ the potential impacts of such noise on welfare and phenotype have not been explored to date. To our knowledge, only two studies have explicitly considered ultrasonic noise when examining the impact of noise on mice, and neither tested the impact of ultrasonic frequencies in isolation of those audible to humans. ^39^^,^^44^

We used two commercially available systems to examine potential ultrasonic noise sources at the Mary Lyon Centre at MRC Harwell; the Avisoft Bioacoustics system and the HBK PULSE system. Both systems detected the continuous ultrasonic (e.g. from the ceiling lights) and metal-on-metal impact noises; however, the increased sensitivity and gain used for the microphone of the Avisoft system introduced artefacts. If we had not used both systems to investigate the noise made by walkie-talkie radios in this instance, we would have not discovered this was an artefact. It is advised that measures are taken to minimise exposure if a noise is identified, either audible or ultrasonic, which could therefore potentially unnecessarily impact resources. It is recommended that detected ultrasonic noise is investigated thoroughly and measured accurately where possible.

Whilst both systems could be used to detect ultrasonic noise, in our study, only the PULSE system could be used to obtain accurate and reliable measures of frequency and sound pressure level. The Avisoft system in the configuration used here could only provide relative measures. A calibrator could be used to improve the accuracy of measurements obtained from the Avisoft system, but due to the flat frequency response of the type 4939 microphone, the PULSE system would nonetheless remain the most suitable system in this area. Thus, whilst the Avisoft system, and likely other ultrasonic microphones intended for a similar purpose, could be used to identify ultrasonic noise sources, accurate measurement equipment such as the PULSE system is preferable for precisely quantifying frequency content and sound pressure level. The PULSE system is, however, far less accessible (>£20k) than ultrasonic microphones typically used to record USVs (price range ~-£250–~£5k). Other options such as the MEMS-based AudioMoth full spectrum logger may be useful as an alternative option to the more RF-sensitive, high-gain ultrasonic condenser microphones such as the CM16, for routine detection or periodical monitoring of ultrasonic (and audible) noise; however, they also have the disadvantage of not being easy to calibrate for measurement over the required frequency range.

If animal facilities possess the equipment to record USVs we suggest it would be beneficial for them to perform an in-house investigation to identify potential sources of noise. The frequency range of this investigation should be determined according to the reported audible range of the species contained within. Our findings also indicate that proper measurement of detected noise sources is essential when looking at mitigating measures. Equipment to perform these investigations is available to hire, however, operation and interpretation of data may require specialist consultation.

Ultrasonic noise detected in this study could potentially impact mouse welfare and phenotype. This is an area that certainly warrants further investigation, as actions to mitigate ultrasonic noise are most likely to occur only when a detrimental effect has been identified. However, we strongly feel that, especially given the importance of mouse USVs in social communication ^34^^–^^38^ and evidence of how impactful other frequencies can be to welfare, ^15^^,^^21^^,^^22^ steps should be taken to minimise noise wherever possible. Although harder to detect, ultrasonic noise is comparably easier to attenuate by shielding than noise with a lower frequency content. We observed that ultrasonic noise from the ceiling light was not detected by either system from inside the IVC, so the cage itself will probably protect mice from low level ultrasonic sources. However, as the aim of this type of investigation is to find noise sources that specifically affect the animals, measurements should always be taken from both inside a modified cage and outside to confirm this. Actions to minimise ultrasonic noise that is high-level and can be detected within a sealed IVC in particular should be a priority, as mice will be exposed under normal housing conditions. Such noise could conceivably hinder social communication and impact other traits related to health, welfare and behaviour even when mice are not exposed to any other form of stress outside of routine husbandry procedures.

Whilst noises detected outside of the IVC may not be of a high level, the ultrasonic frequencies will normally be readily attenuated by the cage. This may make mice more sensitive to these frequencies when they are exposed. The presence of ultrasonic noise whilst performing behavioural testing could be particularly impactful; the mice are infrequently exposed to the noise, and sources may vary from room to room. Steps should be taken to investigate and minimise such noises in experimental areas to reduce potential negative effects on the reliability of data. Mitigation of impact noise, such as that produced in copulatory plug checking, may be relatively simple. As shown in this study, it can be substantially controlled with cost-effective and easy-to-implement modifications; in some cases simple procedural refinements may be sufficient. As mice can also hear the sound very clearly inside the cage, this should be a priority.

Current guidelines for noise surveys in animal facilities recommend that multiple frequencies are considered, and typically mention that mice can hear ultrasonic frequencies. ^6^^–^^8^^,^^10^ However, the guidelines do not make specific recommendations regarding the range of frequencies to consider, the ideal length of surveys or the distance from potential noise sources that measurements should be performed. This lack of specificity prevents standardisation, meaning that mice in different facilities may have vastly different acoustic environments. Ideally, noise surveys should consider all frequencies audible to the animals housed in that facility but, whilst a good starting point, this alone could be insufficient to minimise exposure to extraneous noise. For example, facilities that conduct longer sound surveys are more likely to detect periodic noises, such as those from equipment that is not constantly running or procedures that are conducted relatively infrequently. Such facilities are thus more likely to act to minimise exposure to periodic noise. Further, since ultrasonic sound is highly directional and travels a shorter distance than lower frequency sound, noise measurements undertaken in one part of a room may fail to detect ultrasonic noise that mice housed elsewhere in that room are exposed to.

The potential consequences of exposure to ultrasonic noise have not been studied to date but could be profound. Mice use USVs for social communication, ^37^^,^^38^ and whilst they are able to distinguish between USVs and noise, ^45^^,^^46^ it is perceivable that ultrasonic noise could hinder social communication. This could be detrimental to welfare by leading to increased levels of aggression or simply preventing normal social behaviour. Whilst unlikely under IVC housing conditions, exposure of higher levels of ultrasonic noise could also lead to hearing loss, as with exposure to noise with lower frequencies, ^19^^,^^20^ which could prevent mice from detecting USVs, at least at particular frequencies. Further, human studies have shown that exposure to audible noise, particularly at high levels, can affect multiple traits, including stress, cardiovascular function and anxiety. ^25^^,^^43^ As ultrasonic frequencies are audible to mice, it follows that exposure to them may have similar effects. Any such effect could be detrimental to welfare, increase mortality, reduce productivity and/or confound studies, particularly those which require phenotyping outside of the home cage. Exposure to ultrasonic noise could therefore be a source of unnecessary stress for animals in animal housing facilities and could lead to the need for greater numbers of animals in studies for sufficient statistical power, and because of the lack of standardisation of sound surveys, this could lead to reduced study reproducibility. These consequences are in direct conflict with the principles of the 3Rs, aimed at performing more humane animal research by minimizing the pain, suffering, distress or lasting harm experienced by the animals in research, via “Replacement, Reduction and Refinement” and steps taken to reduce noise exposure, audible or otherwise would constitute a refinement. ^41^

## Conclusions

Multiple systems, including those discussed here, can be used to identify sources of ultrasonic noise. When selecting a system to use, it is important to consider the relative advantages and disadvantages of each. Sound analysis systems such as the PULSE system, when combined with a suitable calibrated microphone, can provide accurate measurements of sound pressure level and frequency. Recordings from ultrasonic microphones like the Avisoft and AudioMoth systems used in this study can also be employed to detect ultrasonic frequencies that are inaudible to humans; as long as gain settings and processing remain consistent, they could also be useful for comparing relative levels as an initial investigation, and can be more accessible. Furthermore, in some cases the ability to visualise the spectrogram of these recordings with high temporal resolution could be helpful in identifying the source of an ultrasonic noise. It is also important to remember that measurement and detection are distinct when selecting a system. Thus, if the purpose of testing is to examine noise sources that affect animals and reduce the chance of exposure, and the hire or acquisition of accurate measurement equipment is not currently financially possible, using a cheaper method of detection is likely more important than measuring exact levels. However, as with workplace surveys of audible noise, accurate measurement is important to assess exposure and environmental impact. If the current guidelines surrounding the acoustic environment of the animals were to be changed to include ultrasonic frequencies, accurate measurement would almost certainly be required.

Due to the nature of scientific research involving animals, animal house staff normally spend more time in the facility with the animals than the researchers. As such, they are arguably best placed to identify potential sources or causes of noise (i.e. through noticing changes in animal behaviour). Animal staff are also dedicated to maintaining the highest standards of welfare for the animals in their care, and as such they should be made aware of the potential exposure of mice to ultrasonic noise, which could potentially have adverse effects. Attention should also be drawn to the ultrasonic frequency content of some impact noises, in particular those involving metal such as forceps or wire cage tops. Any simple procedural or material changes that would minimise this noise will only benefit the animals.

It would be a positive step for animal facilities to be more aware of noise, audible and ultrasonic, and try to implement local policies for identifying and mitigating any noise sources relevant to the species housed within. We would advise that noise surveys should be sufficiently long to identify periodic noise (e.g. from equipment that is not in constant use) and tests should be undertaken at the minimum distance that the equipment will be used from mice (both inside and outside of an IVC, where this is the housing used). A particular emphasis should be placed on assessing areas with the highest potential of exposure, such as phenotyping rooms where mice are frequently handled outside of their home-cage. Ideally, all new equipment would be tested as a potential noise source when it is first introduced into an animal housing facility, so that appropriate precautions can be taken to minimise noise exposure. We also recommend that studies are undertaken to evaluate the impact of ultrasonic noise on mice. Unless there is conclusive evidence that there is no detrimental effect, steps should be taken to minimise ultrasonic noise wherever possible; this can usually be achieved simply at no or minimal cost either by operational changes or simple shielding methods; however sources must first be identified. We encourage scientists to disclose sound profiles of their facilities and the testing environment in publications. This would not only enable the potential impact to be assessed at a later date, but also raise awareness of the issue.

## Data availability

### Underlying data

Harvard Dataverse: Investigating Audible and Ultrasonic Noise in Modern Animal Facilities. https://doi.org/10.7910/DVN/QSCYBS

This project contains the following underlying data:

- Audio moth away from speaker.wav (recording of the walkie-talkie operation not in proximity to a speaker using the AudioMoth)
- Audio moth near speaker.wav (recording of the walkie-talkie operation in close proximity to a speaker using the AudioMoth)
- Avisoft.wav (original recording of walkie-talkie being operated using the Avisoft system)

Data are available under the terms of the Creative Commons Zero “No rights reserved” data waiver (CC0 1.0 Public domain dedication).
