# Supplementary material for: Investigating audible and ultrasonic noise in modern animal facilities
Source: F1000Res. 2022 Jun 14;11:651. [Version 1] doi: 10.12688/f1000research.111170.1 (PMC9334837; doi:10.12688/f1000research.111170.1)
Supplement: Peer review history

# Peer review: Reviewer response for version 1

**DOI:** 10.5256/f1000research.122863.r140878

**Published:** 2022-07-28

**Authors:** Leonardo Restivo

**Affiliations:** 1 Neuro-Behavioral Analysis Unit (Neuro-BAU), Department of Fundamental Neuroscience, Faculty of Biology and Medicine, University of Lausanne, Lausanne, Switzerland

Biomedical scientists working with research animals are becoming increasingly aware of factors affecting animal welfare and experimental procedures. Animal facilities must follow strict guidelines determining acceptable ranges of potentially disruptive factors such as light, noise, temperature, and humidity. Currently, there are no guidelines dictating standards of acceptable noise range in the ultrasonic domain for rodent facilities. Knowing the negative impact that noise may exert on rodents' welfare and experimental procedures, this manuscript sets out to implement novel and refined techniques to assess sources of ultrasonic and audible noise commonly found in rodent housing facilities. The authors present a detailed analysis of the benefits and caveats of the two systems deployed to capture the sources of ultrasonic noise.

 The manuscript is a significant step toward the definition of a "systemic" approach to biomedical research. The "experiment" is commonly defined as the interval of time during which the subject is under direct experimentation (e.g., application of selected assays), often neglecting that living creatures experience time as a continuum. Therefore, factors affecting animal welfare outside of the limited time covered by the experimental procedure are bound to impact the quality of the data collected throughout the experiment. For this reason, it is critical to design guidelines that regulate factors present in the housing facility (e.g., light, temperature, humidity, noise), including sources outside the perceptual range of human operators (e.g., ultrasonic noise). The authors performed ultrasonic noise analysis both outside and inside the home cage (IVC), leading to the critical finding that some noise sources can reach mice inside their air-sealed home cage. Critically, the authors show that simple and inexpensive procedures can be deployed to minimize ultrasonic noise disruptions. As correctly stated in the manuscript, we do not know yet the consequences of exposure to ultrasonic noise. Therefore, this manuscript is also a call to action for animal welfare scientists to further investigate the effects of ultrasonic noise on rodents. Nonetheless, I support the authors when they state that in the absence of conclusive evidence, all animal facilities must be encouraged to check their noise landscapes, including ultrasonic noise, and to take action to eliminate or dampen the sources of this potentially disruptive noise.

Is the work clearly and accurately presented and does it cite the current literature?

Yes

If applicable, is the statistical analysis and its interpretation appropriate?

Not applicable

Are all the source data underlying the results available to ensure full reproducibility?

Yes

Is the study design appropriate and is the work technically sound?

Yes

Are the conclusions drawn adequately supported by the results?

Yes

Are sufficient details of methods and analysis provided to allow replication by others?

Yes

Reviewer Expertise:

Behavioural Neuroscience

I confirm that I have read this submission and believe that I have an appropriate level of expertise to confirm that it is of an acceptable scientific standard.

# Peer review: Reviewer response for version 1

**DOI:** 10.5256/f1000research.122863.r142997

**Published:** 2022-07-18

**Authors:** Markus Brielmeier

**Affiliations:** 1 Core Facility Laboratory Animal Services, Helmholtz Munich, Research Center for Environmental Health, Neuherberg, Germany; 2 Veterinary Faculty, Ludwig Maximilians Universität München, Munich, Germany

The manuscript shows how to measure sound and ultrasound in an experimental animal facility. The advantages and disadvantages of 2 measurement systems available on the market are reported and possible artifact sources are discussed.

 I agree with the authors that too little attention is paid to the disturbance of laboratory animals by inaudible ultrasound. As mice communicate with ultrasound, it is likely that they are disturbed by ultrasound at high sound levels. The authors suggest that suitable equipment should be used to identify and, if possible, attenuate the sources of ultrasound. Using the example of impact noises (e.g. noises made by tweezers during transfer), they show that simple measures can be taken to attenuate the sound. Although mice are almost only kept in individually ventilated cages and the plastic attenuates ultrasound very well, the authors show that ultrasound at high levels does reach into the cage. Moreover, most of the tests still take place outside the protective cage and there the mice are exposed to the various ultrasound sources. If ultrasound affects phenotypes, it will contribute to de-standardization, which is unsatisfactory from a scientific point of view.

Is the work clearly and accurately presented and does it cite the current literature?

Yes

If applicable, is the statistical analysis and its interpretation appropriate?

Not applicable

Are all the source data underlying the results available to ensure full reproducibility?

Yes

Is the study design appropriate and is the work technically sound?

Yes

Are the conclusions drawn adequately supported by the results?

Yes

Are sufficient details of methods and analysis provided to allow replication by others?

Yes

Reviewer Expertise:

Laboratory Animal Science, Microbiology, Sound and Vibration measurement, home cage technologies

I confirm that I have read this submission and believe that I have an appropriate level of expertise to confirm that it is of an acceptable scientific standard.
